# A Two-week, Hands-on Educational Program for Primary Care Pediatricians Aimed at Equalization of Pediatric Allergy Practice across Institutions and Regions

**DOI:** 10.31662/jmaj.2024-0127

**Published:** 2024-10-07

**Authors:** Fumi Ishikawa, Tatsuki Fukuie, Yasuaki Matsumoto, Daichi Suzuki, Kotaro Umezawa, Kazuma Takada, Seiko Hirai, Kenji Toyokuni, Mayako Saito-Abe, Miori Sato, Yumiko Miyaji, Shigenori Kabashima, Kiwako Yamamoto-Hanada, Kohta Suzuki, Yukihiro Ohya

**Affiliations:** 1 Allergy Center, National Center for Child Health and Development, Tokyo, Japan; 2 Department of Health and Psychosocial Medicine, Aichi Medical University of Medicine, Aichi, Japan

**Keywords:** Allergy, Immunology, Community medicine, Primary care, Pediatrics

## Abstract

**Introduction:**

Similar to other countries, in Japan, the demand for primary care pediatricians has increased due to the surge in pediatric allergic diseases, and with the change in a paradigm shift regarding the prevention of pediatric allergic disease in the last 20 years, they have had an increased need for retraining. To offer better support to children and their caregivers, educational needs for bridging the gap between knowledge and practice must be met. Therefore, we developed an educational program including practical and interactive approaches for pediatricians in 2012.

**Methods:**

To evaluate the effectiveness of a 2-week program, behavioral changes, knowledge and skill improvements in clinical practice, and the satisfaction level of participants before and after the course were investigated. Kirkpatrick’s four levels of training evaluation were employed to assess the educational effect. Seven years (April 2014 to March 2021) worth of results were assessed.

**Results:**

A total of 65 pediatricians voluntarily participated in the program. Most of them were <40 years old and came from various regions of Japan. Results of pretraining and posttraining questionnaires in terms of their knowledge and skills on a four-point scale revealed significant improvements. Participants also reported their behavioral changes after 6 months of the course and evaluated the program’s practicality. Each participant set new goals to be achieved in 6 months, and 36 (76.6%) of them set objectives for implementing oral food challenge tests.

**Conclusions:**

The results revealed that the program not only enhanced their knowledge and skills for practice but also changed their behaviors toward clinical practice. In pediatric allergy, where community primary pediatricians have important roles to play, such an educational program should be further developed.

## Introduction

For decades, the prevalence of pediatric allergic diseases has increased worldwide, and this problem has worsened the concerns of many children and their caregivers ^(1)^. Thus, more primary care clinicians have been asked to appropriately manage their diseases ^(2), (3)^. In Japan, pediatric allergists have been unevenly distributed, and as in other countries, community primary care pediatricians have been in demand for accurate diagnostic and therapeutic roles ^(4), (5), (6)^.

Additionally, since the consecutive publication of the idea of appropriate intervention for infantile atopic dermatitis (AD) and early food introduction based on the “dual-allergen exposure hypothesis” and supporting research from 2000 to 2010s, the understanding of allergic diseases has significantly advanced ^(7), (8), (9)^. A major paradigm shift and therapeutic innovation have taken place in this area.

Even though medical care and bridging gaps in knowledge and skills in practice in this field need to be enhanced ^(10), (11)^, pediatricians have had few opportunities to obtain sufficient education to update them ^(12), (13)^. An opportunity to gain knowledge may at least be available, but it is not sufficient to bring about behavioral change in the practice.

To meet the needs, in 2012, we developed a 2-week educational program for pediatricians to manage food allergies (FAs). Reportedly, the effectiveness of this program during the first and second terms in 2013-2014 has brought about changes in the knowledge, skills, and behaviors of the participants ^(14)^. In 2019, new contents were added to the program, and it was renamed to “short-term focused education and training program for pediatric allergy practice,” in accordance with basic guidelines in the 2017 Allergy Disease Control Act (enforced in 2015), which required the promotion of equalization on allergy practice and education in Japan. Therefore, in this study, the effectiveness of this continuing education program in the last 7 years following our first report was evaluated ^(14)^.

## Materials and Methods

### Study design

In this study, the improvement in the level of knowledge/skills and practice behaviors of participants was investigated through the program performed between April 2014 and March 2021.

### Participants

The course was intended for general pediatricians with approximately 5-20 years of experience who were willing to obtain the latest pediatric allergy knowledge and skills. Information regarding the program was spread annually through leaflets, mailing lists, and the website of our institute for participant recruitment. In 2019, participants from “base hospitals of prefectures,” which were newly placed and played a central role in the medical practice for allergic diseases under the Allergy Disease Control Act, were invited to actively participate in this study.

### Educational program

The original program established 22 specific knowledge/skill objectives that describe measurable changes via educational intervention. Considering that the control of AD is necessary for FA management, two behavioral changes: “intervention for AD with appropriate skin care and topical therapy” and “stopping excessive elimination dietary guidance” were set as final goals (items for behavioral evaluation). Based on these goals, seven behavioral objectives were set to be achieved within 6 months. Moreover, at least three behavioral objectives were set within 6 months by each participant freely at the end of the course. In 2019, the contents of AD, asthma, and allergic rhinitis (AR) were added to ensure the management of coexisting allergic diseases, and an additional 36 skill/knowledge objectives and 14 behavioral objectives were set. The textbook was edited corresponding to each objective with reference to international guidelines, Japanese guidelines, specialized books, websites of international institutes, and expertise of important literature in professional journals.

The program methods involved attendance in the examination or treatment in daily practice, hands-on experience, participation in patient educational programs, case-based learning, lectures, and textbook discussions conducted based on the timetable shown in Table 1. From the program initiation, we highlighted the oral food challenge test (OFC) and incorporated it into the daily plan. To meet participants’ diverse needs and to keep being practical and having interactive approaches, an advisor from our staff members assisted them with schedule management and questioning, and tutors gave lectures for each objective.

**Table 1.**
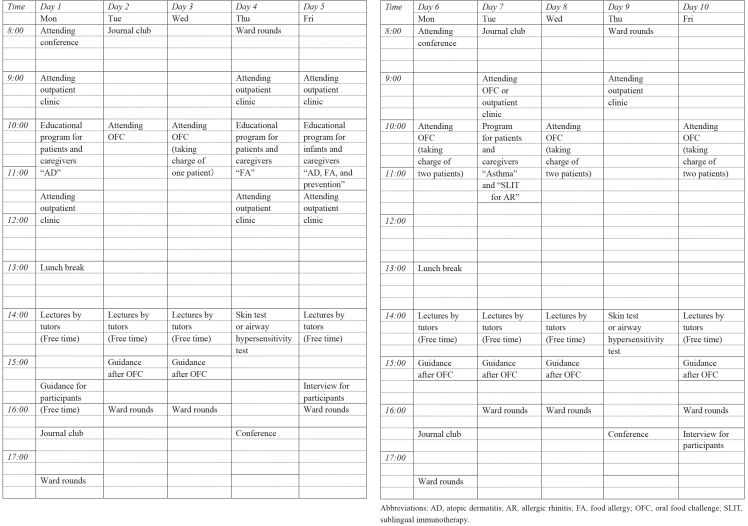
Timetable.

### Data collection and statistical analysis

The effectiveness of the program was evaluated by analyzing questionnaires on the basis of Kirkpatrick’s four levels of training evaluation ^(15)^, and only the results with both before and after responses were analyzed. This model comprises levels 1-4, namely, “reaction,” “learning,” “behavior,” and “results,” respectively. As for level 1 “reaction,” the extent of participants’ satisfaction with the contents of the training program, volume, methods, and support system was evaluated based on a four-point Likert scale, ranging from unsatisfied (1) to highly satisfied (4) when they completed the course. As for level 2 “learning,” acquisition levels were evaluated using a four-point Likert scale, ranging from no (1) to yes (4) at the beginning and completion of the course. As for level 3 “behavior,” behavioral changes in participants’ practice were evaluated as no (0) or yes (1) at the beginning and 6 months after the course. Moreover, behavioral objectives to be achieved after 6 months were freely set by the participants. The answers to the questionnaire and achievement rates of individual goals 6 months after the educational program were collected via postal delivery. The supplementary data (Supplementary Table 1, Supplementary Table 2, Supplementary Table 3, Supplementary Table 4) list the actual question items. The additional behavioral objectives were classified into nine categories, namely, “A. skin prick test (SPT),” “B. OFC,” “C. Dietary guidance,” “D. Management of acute reaction,” “E. Skin care, management of AD,” “F. Prevention of FA, intervention in early infancy,” “G. General matter of FA,” “H. Management of asthma,” and “I. Sublingual immunotherapy (SLIT) for AR” (H. and I. are in the renewed program). Intervention procedures to carry out these plans described freely were also classified into six groups, namely, “a. Examination or treatment as practice,” “b. Education for patients,” “c. Education for citizens,” “d. Education for medical staff / multidisciplinary medicine,” “e. Cooperation between hospital and clinic,” and “f. Establishing manual or system.”

All statistical analyses were conducted using EZR software (Saitama Medical Center, Jichi Medical University, Saitama, Japan), which is a graphical user interface for R (The R Foundation for Statistical Computing, Vienna, Austria). To evaluate changes in participants’ “acquisition of knowledge and skills,” the Wilcoxon signed-rank test was employed for “behavioral changes in the participants’ practice” in the binominal test, following Bonferroni’s correction.

### Ethical considerations

This study was conducted according to the principles of the Declaration of Helsinki of 1965 (as revised in Brazil 2013). Participants provided written consent to collect, analyze, and publish the results without personal information. Anonymity was preserved using methods approved by the Ethics Committee. The institutional review board of the National Center for Child Health and Development approved this study (No. 2019-014).

## Results

### Participant backgrounds

A total of 65 pediatricians participated in the program in the third (2014-2015), fourth (2015-2016), fifth (2016-2017), sixth (2017-2018), seventh (2018-2019), eighth (2019-2020), and ninth (2020-2021) terms, with 12, 9, 10, 10, 8, 15, and 1 participant, respectively. Despite the expansion of the program’s role, between 2020 and 2021, numerous applicants had to cancel participation because of the coronavirus disease 2019 pandemic.

Table 2 presents the backgrounds of the participants. Of the 65 participants, 50 (76.9%) were under 40 years old. Fifty (76.9%) were pediatric specialists certificated by the Japan Pediatrics Society. Two physicians participated through childcare leave. Forty-six of them were working in large hospitals with more than 200 beds, six in hospitals with less than 200 beds, and seven in clinics. Their workplace covered 24 of 47 prefectures in Japan. Furthermore, 31 (47.7%) participants were from the Kanto area including Tokyo, the remaining from other areas, and one from overseas. Between 2019 and 2021, nine participants were from the base hospitals of the prefectures.

**Table 2.** Participant Characteristics.

Age (years), median (interquartile range) (n = 64)		36 (32-40)
Male, (%) (n = 65)		33 (50.7)
Experience as a physician (years), median (interquartile range) (n = 65)		10 (8-14.5)
Workplace (district), No. (%) (n = 65)	Hokkaido	3 (4.6)
	Tohoku	7 (10.8)
	Chubu	6 (9.2)
	Kanto	31 (47.7)
	Kansai	9 (13.8)
	Chugoku and Shikoku	6 (9.2)
	Kyushu and Okinawa	2 (3.1)
	Overseas	1 (1.5)
Workplace (facility), No. (%) (n = 65)	Hospitals with ≥200 beds	46 (70.1)
	Hospitals with <200 beds	6 (9.2)
	Clinic	7 (10.8)
	No answer/parental leave	6 (9.2)
From the central base hospital of prefectures^†^ (n = 16)		9 (56.3)
Pediatric specialists, No. (%) (n = 65)		50 (76.9)
Allergy specialists, No. (%) (n = 65)		4 (6.2)
Number of patients undergoing OFC in participant’s facility per month, No. (%) (n =55^‡^)	0^§^	23 (41.8)
	1-9	19 (34.5)
	≥10	13 (23.6)
Number of patients undergoing SPT in participant’s facility per month, No. (%) (n = 55^‡^)	0	29 (52.7)
	1-4	14 (25.4)
	≥5	10 (18.1)
	No answer	2 (3.6)
Knowing the “dual-allergen exposure hypothesis,” No. (%) (n = 45^‡^)	Yes	35 (77.8)
	No	10 (22.2)

^†^Started collecting data after 2019. ^‡^In the fifth term (2015-2016), these questions were not asked. ^§^Including answers of participants who were on leave. Abbreviations: OFC, oral food challenge test; SPT, skin prick test.

Before attending the course, 23 of 55 respondents (41.8%) had not conducted OFC in their facility, and 19 (34.5%) had performed OFC on less than 10 patients per month. Thirteen respondents who answered performing OFC to more than 10 patients per month were all employees of large hospitals. Of 45 respondents, 35 (77.8%) answered that they knew the “dual-allergen exposure hypothesis” and 10 (22.2%) did not.

### Evaluation of reaction (Kirkpatrick level 1)

**Figure 1.**
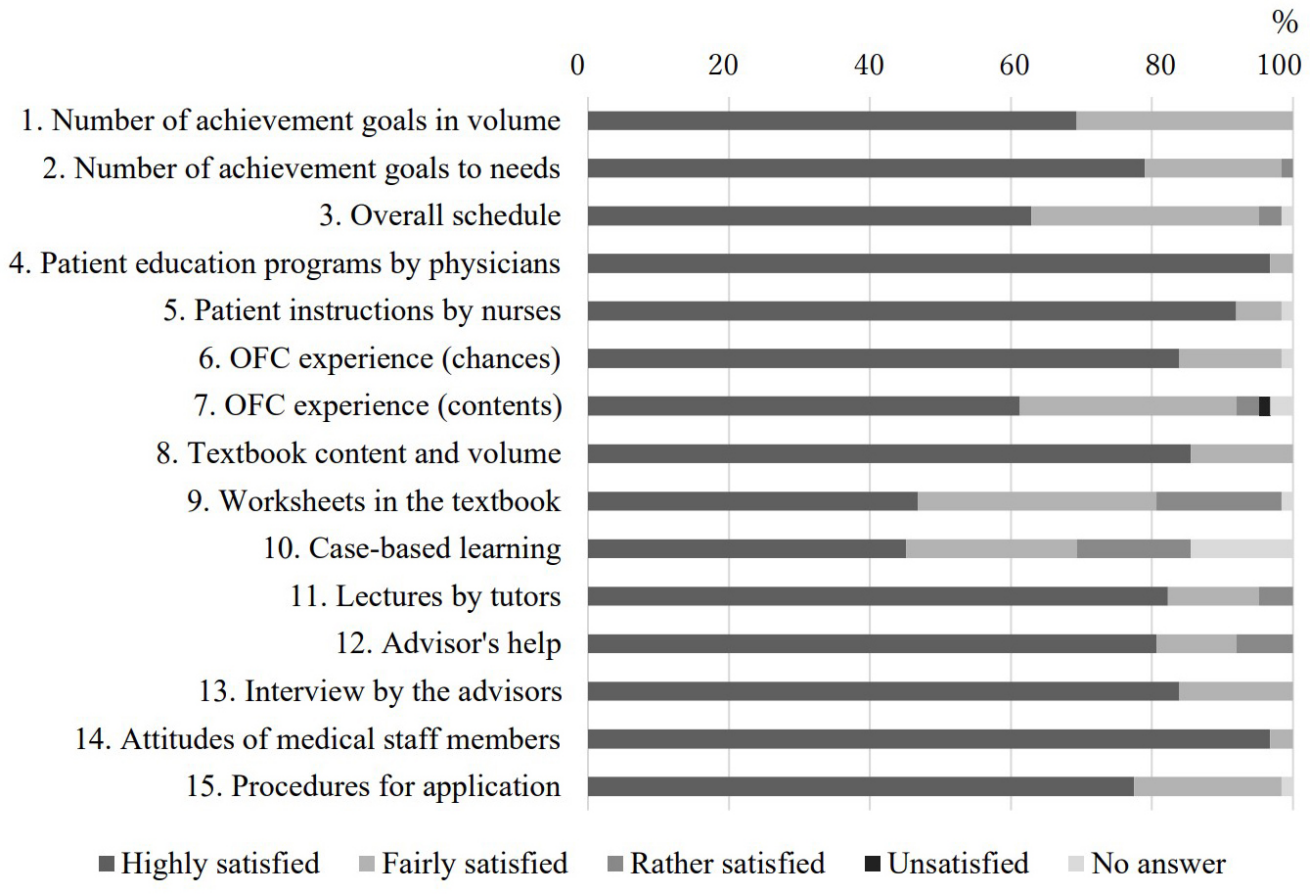
Course evaluations by the participants Answers for patient education programs by physicians (Item No. 4) and instructions by nurses (Item No. 5) were found to be highly meaningful for the participants. Abbreviations: OFC, oral food challenge test.

Figure 1 shows the results of posttraining questionnaires for the satisfaction (course evaluations) of 62 participants. Scores for items to be studied in clinical practice, such as patient education programs by physicians (Item No. 4), instructions by nurses (Item No. 5), and experiences of OFC (Item Nos. 6 and 7) were high. Scores for items on the text, such as case-based learning and worksheets for taking notes (Item Nos. 9 and 10) were relatively low.

### Evaluation of knowledge and skills (Kirkpatrick level 2)

Table 3 presents the pre- and post-training questionnaires regarding knowledge and skill acquisition. The answers from 47 participants between the third and seventh terms and all 16 participants between the eighth and ninth terms were analyzed.

**Table 3.** Evaluation of Knowledge and Skills.

Items related to knowledge and skills (third to seventh terms)		Mean achievement score (SD)		*P* value	Items related to knowledge and skills (eighth to ninth terms)		Mean achievement score (SD)		*P* value
		Pre-training	Post-training				Pre-training	Post-training	
[Diagnosis and evaluation of food allergy]									
1	Diagnosis of immediate allergic response	2.74 (0.64)	3.70 (0.46)	<0.001*	1	Diagnosis of immediate allergic response	2.81 (0.91)	3.44 (0.73)	0.010
2	Explanation of the differences in diagnostic methods for FA	2.36 (0.79)	3.59 (0.53)	<0.001*	2	Explanation of the differences in diagnostic methods for FA	2.69 (0.87)	3.56 (0.63)	0.002
3	Interpretation of SPT results	1.48 (0.80)	3.38 (0.64)	<0.001*	3	Interpretation of SPT results	2.13 (0.89)	3.44 (0.51)	0.001*
					4	Diagnosis based on allergen components	2.25 (0.86)	3.44 (0.51)	<0.001*
[Oral food challenge test]									
4	Explanation and giving informed consent for OFC	2.39 (1.08)	3.70 (0.51)	<0.001*	5	Explanation and giving informed consent for OFC	2.38 (0.89)	3.13 (0.89)	0.031
5	Prescription and treatment instructions for OFC	2.09 (1.02)	3.60 (0.54)	<0.001*	6	Prescription and treatment instructions for OFC	2.00 (0.97)	2.94 (0.77)	0.003
6	Supervision of preparation by the medical staff for OFC	2.09 (1.04)	3.66 (0.52)	<0.001*	7	Supervision of preparation by the medical staff for OFC	2.38 (1.47)	3.38 (0.72)	0.005
7	Preparation of the test food and medical devices for OFC	1.66 (0.81)	3.60 (0.54)	<0.001*	8	Preparation of test food and medical devices for OFC	2.19 (1.05)	3.25 (0.77)	0.002
8	Conducting OFC for a patient	1.66 (0.81)	3.53 (0.55)	<0.001*	9	Conducting OFC for a patient	2.38 (1.09)	3.50 (0.73)	0.002
					10	Conducting double-blind food challenge tests	1.81 (0.91)	2.31 (0.87)	0.015
[Dietary guidance]									
9	Listing notes in reintroducing foods for patients at low risk	2.40 (0.88)	3.49 (0.59)	<0.001*	11	Listing notes in reintroducing foods for patients at low risk	2.43 (0.89)	3.43 (0.63)	<0.001*
10	Listing notes for sensitized patients	2.15 (0.81)	3.45 (0.54)	<0.001*	12	Listing notes for sensitized patients	2.38 (0.89)	3.31 (0.60)	0.002
11	Guidance on partial introduction or reintroduction	1.87 (0.80)	3.12 (0.65)	<0.001*	13	Guidance on partial introduction or reintroduction	2.31 (0.87)	3.19 (0.91)	0.002
[Emergency treatment]									
12	Immediate allergic response and emergency action	2.77 (0.79)	3.83 (0.38)	<0.001*	14	Immediate allergic response and emergency action	2.94 (0.77)	3.56 (0.63)	0.002
13	Explanation of the efficacy and dosage of AAI	2.68 (0.81)	3.78 (0.42)	<0.001*	15	Explanation of the efficacy and dosage of AAI	3.00 (0.73)	3.69 (0.60)	0.003
14	Rules to the prescription of AAI	2.32 (0.98)	3.54 (0.66)	<0.001*	16	Rules about the prescription of AAI	2.53 (0.99)	3.31 (0.87)	0.004
15	Usage instructions for AAI	2.68 (0.91)	3.81 (0.45)	<0.001*	17	Usage instructions for AAI	3.38 (0.72)	3.69 (0.60)	0.037
[Management of atopic dermatitis]									
16	Description of the loss of skin barrier function, aggravating factors of AD	2.51 (0.80)	3.74 (0.44)	<0.001*	18	Diagnostic criteria of AD	2.56 (0.81)	3.56 (0.51)	0.003
17	Instructing techniques of skincare	2.44 (0.69)	3.79 (0.41)	<0.001*	19	Explanation of the loss of skin barrier function in AD	2.44 (0.81)	3.44 (0.51)	0.002
18	Explanation of the side effects of topical corticosteroids	2.77 (0.60)	3.81 (0.40)	<0.001*	20	Evaluation of the severity of AD	2.25 (0.86)	3.06 (0.57)	0.007
19	Avoiding side effects of topical corticosteroids	2.34 (0.70)	3.72 (0.45)	<0.001*	21	Instructing techniques of skincare	2.63 (0.81)	3.53 (0.64)	0.004
20	Idea of proactive treatment for AD	2.47 (0.95)	3.83 (0.38)	<0.001*	22	Avoiding side effects of topical corticosteroids	2.50 (0.82)	3.53 (0.52)	0.002
					23	Idea of proactive treatment for AD	2.63 (0.96)	3.60 (0.51)	0.005
					24	Exacerbation factors of atopic dermatitis	2.75 (0.68)	3.53 (0.52)	0.004
[Allergy prevention concept]									
21	Knowledge of the theory “dual-allergen exposure hypothesis”	2.30 (0.93)	3.77 (0.43)	<0.001*	25	Knowledge of the theory “dual-allergen exposure hypothesis”	2.00 (0.89)	3.33 (0.49)	<0.001*
[Management of asthma]									
					26	Diagnostic criteria of asthma	2.63 (0.72)	3.40 (0.51)	0.002
					27	Evaluation of the severity and control status of asthma	2.75 (0.68)	3.47 (0.52)	0.002
					28	Exacerbation factors of asthma	2.88 (0.62)	3.60 (0.50)	0.002
					29	Evaluation of pulmonary function	2.25 (1.00)	3.33 (0.62)	0.002
					30	Evaluation of fractional exhaled nitric oxide (FeNO)	2.06 (0.93)	2.93 (0.70)	0.002
					31	Conducting airway sensitivity tests	1.19 (0.40)	2.07 (0.70)	0.002
					32	Long-term management of asthma	2.63 (0.72)	3.40 (0.51)	0.006
					33	Guidance on the lifestyle and household environment for preventing asthma exacerbation	2.50 (0.89)	3.40 (0.74)	0.006
					34	Inhalation device	2.56 (1.03)	3.40 (0.63)	0.009
					35	Action plans for acute asthma exacerbation	2.94 (0.85)	3.60 (0.51)	0.005
[Allergic rhinitis]									
					36	Sublingual immunotherapy	2.06 (1.12)	3.20 (0.68)	0.005
[Patient education]									
22	Educational interventions for patients	2.11 (0.68)	3.43 (0.54)	<0.001*

Abbreviations: AAI, adrenaline auto-injector; AD, atopic dermatitis; FA, food allergy; OFC, oral food challenge test; SPT, skin prick test. Note: For the third to seventh terms, *P* < 0.0023 is considered statistically significant and for eighth to ninth terms, and *P* < 0.0014 is considered statistically significant.

Between the third and seventh terms, every score of participants’ knowledge and skills for FA increased significantly after training. Specifically, great enhancements were noted in the following items on preparation and organization of OFC: Item No. 4, 2.39 ± 1.08 (mean ± SD) to 3.70 ± 0.51, *P* < 0.001; Item No. 5, 2.09 ± 1.02 to 3.60 ± 0.54, *P* < 0.001; Item No. 6, 2.09 ± 1.04 to 3.66 ± 0.54, *P* < 0.001; Item No. 7, 1.66 ± 0.81 to 3.60 ± 0.54, *P* < 0.001; and Item No. 8, 1.66 ± 0.81 to 3.53 ± 0.55, *P* < 0.001. The item on the practice of SPT for FA diagnosis showed significant improvement. Items on AD management (Item Nos. 19, 20, and 21) also depicted increases. In contrast, scores on dietary guidance (Item No. 11) remained relatively low.

Between the eighth and ninth terms, improvements were seen in all items; nevertheless, only two items were statistically significant. Among the newly added items, the achievement rates in Item No. 10 (double-blind food challenge test) and No. 31 (airway sensitivity test) were low. As for how to use the adrenaline auto-injector (AAI), the pretraining achievement rates in the eighth and ninth terms were higher than those in the third to seventh terms.

### Evaluation of behaviors (Kirkpatrick level 3)

Table 4 shows the behavioral changes. Answers of 32 participants between the third and seventh terms and of 12 participants between the eighth and ninth terms, who answered both before and 6 months after training, were analyzed.

**Table 4.** Evaluation of Clinical Practice Behaviors.

	Items related to practice behaviors	Third to seventh terms			Eighth to ninth terms		
		Yes/No			Yes/No		
		Pre-training	Post-training	*P* value	Pre-training	Post-training	*P* value
1	Explanation based on correct test result interpretation for the diagnosis of FA	32/0	32/0	―	12/0	12/0	―
2	Explanation of the importance of immediate treatment of eczema with skincare and prescribed external medicine for patients under a food elimination protocol	24/8	31/1	0.105	12/0	12/0	―
3	Conducting skin tests or OFC for the reintroduction of foods for patients under a food elimination protocol without recent severe immediate reactions	6/26	22/10	<0.001*	4/8	10/2	0.281
4	Practical instructions on skincare and application of external medicine for patients with AD and FA	10/22	31/1	<0.001*	8/4	12/0	0.75
5	Quick interventions for the reintroduction of foods	14/18	26/6	0.001*	9/2	11/0	0.75
6	Confirmation of the improvement of atopic dermatitis after the intervention	6/26	25/7	<0.001*	4/7	8/3	1
7	Giving information on the action plan including AAI	18/14	26/6	0.023*	7/4	9/2	1
8	Explanation of the diagnosis and severity of atopic dermatitis based on diagnostic criteria and severity index				3/8	9/2	0.219
9	Provision of plans and guidance on treatment methods based on proactive remission maintenance therapy of AD				6/6	10/2	0.75
10	Evaluation of exacerbation factors and provision of guidance on lifestyle and household environment for AD				4/8	10/2	0.188
11	Application of self-monitoring tools or questionnaires to evaluate the severity and control status of asthma				3/9	6/6	1
12	Conducting pulmonary function tests, measurement of exhaled nitric oxide concentration, and airway hypersensitivity test to evaluate the severity and control status of asthma				3/8	8/3	0.375
13	Confirmation of the inhalation procedure after inhalation guidance				4/8	8/4	0.75
14	Consideration and application of sublingual immunotherapy for allergic rhinitis				3/8	7/4	0.75

Question items No. 8 to No. 14 were for only eighth and ninth terms. Abbreviations: AAI, adrenaline auto-injector; AD, atopic dermatitis; FA, food allergy; OFC, oral food challenge test. Note: For the third to seventh terms, *P* < 0.0083 is considered statistically significant, and for the eighth to ninth terms, *P* < 0.0042 is considered statistically significant.

Between the third and seventh terms, significant behavioral improvements in six of seven items were shown, except for Item No. 1, which all the respondents achieved already before the course. Improvements were shown in the immediate treatment of comorbid AD (No. 4), and the number of respondents having achieved the goal went up from 10 to 31 (*P* < 0.001). The item on the implementation of diagnostic tests (No. 3) and stopping unnecessary food elimination (No. 5) also improved significantly (6 to 22, *P* < 0.001; 14 to 26, *P* = 0.002, respectively). Between the eighth and ninth terms, we could highlight behavioral improvement in 12 of 14 items; however, they were not statistically significant.

### Plans and goals of respective participants

**Figure 2.**
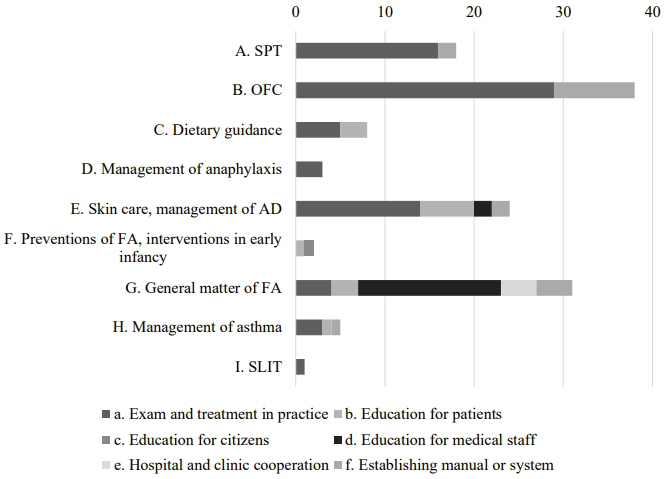
Six-month goals set by each participant after completing the training (multiple answers). The behavioral objectives were classified into nine (A.-I.) categories. H. and I. are present only in the renewed program. The intervention procedures were classified into seven groups (a.-f.). Abbreviations: AD, atopic dermatitis; FA, food allergy; OFC, oral food challenge test; SLIT, sublingual immunotherapy; SPT, skin prick test.

Figure 2 shows the objectives to be carried out in 6 months, set by respective participants on completion of the course. Forty-seven participants responded with multiple answers. Among these nine categories, 38 (80.8%) respondents set objectives for the implementation of OFC. As intervention procedures, 29 planned to introduce OFC into their practice (B-a), and nine planned to establish a manual or system (B-f). The mean achievement rates in 6 months were 67.1% for introducing OFC into their practice (B-a) and 58.8% for establishing a manual or system for OFC (B-f).

Moreover, 16 of 47 (34.0%) respondents planned education for medical staff or sharing information with them (G-d), and the achievement rate in 6 months was 65.5%. Plans for patient education on skin care for AD treatment (E-b) and skin care in daily clinical practice (E-a) displayed high achievement rates in 6 months: 71.7% and 64.4%, respectively.

## Discussion

To obtain our initial purpose, this program enhanced not only participants’ knowledge and skills but also their behaviors in clinical practice after 6 months.

In terms of the program methods, the degree of satisfaction of participants was higher with items with on-site participation than with those using textbooks. In the participants’ opinion, chances to organize OFC, attend patient education programs close at hand, and achieve immediate answers from advisors to their questions were the points evaluated as practical and applicable. Recently, web-based learning systems or brief education tools have been increasingly established as a rapid and effective method in postgraduate medical educational programs, particularly in areas with limited human resources ^(16), (17), (18)^; nonetheless, the classical hands-on training method was highly regarded. We have attempted to maintain the benefit of the framework of the current program and added an e-learning system in 2020 partially for the efficient and comprehensive course of study.

In terms of the program contents, we emphasized two final goals, namely, “intervention for AD with appropriate skin care and topical therapy” and “stopping excessive elimination dietary guidance.” We suggested a long time for OFC experience because we believe that OFC is an essential procedure not only for the standard method to diagnose FA but also for safe food introduction, even for sensitized infants. Safe food introduction is effective for the prevention of FA and gaining tolerance ^(8), (9)^; however, studies have shown various barriers, including the lack of time, space, or staffing for many practitioners to implement OFC ^(19)^. An association was found between the number of OFCs performed in fellowship and those performed in practice in the United States, that is, the lack of experience could be one of the barriers to performing OFC ^(19)^. For participants with a limited number of experience, the program should have been helpful. Notably, 76.6% of the respondents settled on plans to put OFC into their practice after the program.

Although the statistical evaluation was challenging because of the small sample size, advanced achievement rates in knowledge/skill and behavioral items for early intervention for infant’s AD and AAI usage were higher in the eighth to ninth terms than in the third to seventh terms. No different trends were observed in advanced achievement rates between participants from the “base hospital of prefectures” and other participants (data are not shown); thus, changes over time were more strongly suggested.

Some limitations exist in our evaluation procedure. First, the collection of questionnaire results was insufficient, depending on omissions made by the person in charge. Second, because of the limited sample size, particularly in the ninth term, data had insufficient power to show a significant difference, which might result in a type 2 error. Despite this limitation, some results were statistically significant, indicating to be robust. Third, a sampling bias caused by the program setting, that is, 2-week-long and on-site implementation, was noted. Participants with stronger interests might raise the effect of the program. Moreover, responses collected after 6 months from earnest participants might have resulted in indicating higher achievement rates. Fourth, the questionnaire response was based on self-assessment and not collected anonymously. Fifth, Kirkpatrick’s level 4 results (patient outcomes) are difficult to assess. However, certain achievement rates of the goals set by participants after 6 months of the course on system establishment or education for medical staff should contribute to the benefit of patients. Furthermore, since this was a retrospective study, comparing the effectiveness of our method with others to prove the superiority of effectiveness was difficult. We think that evaluating behavioral change was a strength in assessing the effectiveness of this study.

We concluded that our program not only enhanced their knowledge and skills for FA practice but also changed their clinical practice behaviors. Although the method is classical, its importance is realistic, given the recent situation of pediatric FA diagnosis and treatment. As long-term educational effects beyond 6 months have not been evaluated, we would like to consider a prospective, long-term evaluation. In pediatric allergy, where community primary pediatricians require re-education opportunities, such an educational program should be further developed.

## Article Information

### Conflicts of Interest

None

### Sources of Funding

This work was supported by Health and Labour Sciences Research Grant. grant number 201913001B

### Acknowledgement

We would like to express our sincere appreciation to Dr. Yuki Tsumura, Dr. Masami Narita, and co-researchers for developing the program first in 2012, physicians participating in this program and research, their colleagues for supporting participation, and all physicians and medical staff of the Allergy Center at the National Center for Child Health and Development for their assistance in the program. Some of the data were reported at the 123rd annual meeting of Japan Pediatric Society.

### Author Contributions

KY-H, TF, and OY designed the study. FI, TF, YaM, DS, KU, KaT, SH, KeT, MS-A, MS, YuM, SK, and KY-H contributed to data collection. KS made important policy decisions about statistical analysis. FI performed data analysis, and statistical analysis and wrote the manuscript. MS-A, MS, and TF gave important advice on the structure of the paper. All authors read and approved the final manuscript.

### Approval by Institutional Review Board (IRB)

the institutional review boards of the National Center for Child Health and Development (Issue No. 2019-014).

## Supplement

Supplementary Table 1

Supplementary Table 2

Supplementary Table 3

Supplementary Table 4
