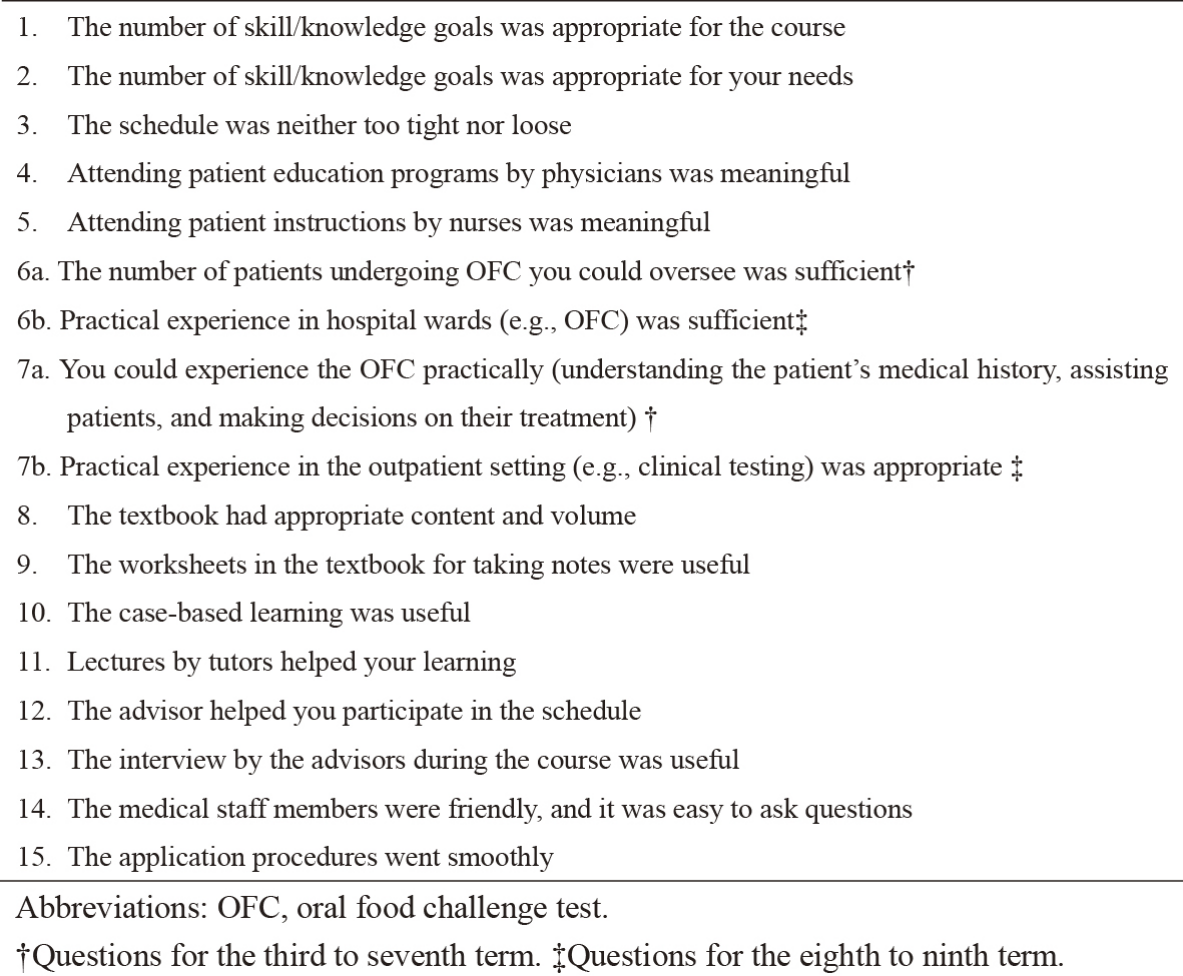# Erratum: 2024;7(4):590-9 (DOI: 10.31662/jmaj.2024-0127)

**DOI:** 10.31662/jmaj.e0002

**Published:** 2025-08-29

**Authors:** Fumi Ishikawa, Tatsuki Fukuie, Yasuaki Matsumoto, Daichi Suzuki, Kotaro Umezawa, Kazuma Takada, Seiko Hirai, Kenji Toyokuni, Mayako Saito-Abe, Miori Sato, Yumiko Miyaji, Shigenori Kabashima, Kiwako Yamamoto-Hanada, Kohta Suzuki, Yukihiro Ohya

**Affiliations:** 1 Allergy Center, National Center for Child Health and Development, Tokyo, Japan; 2 Department of Health and Psychosocial Medicine, Aichi Medical University of Medicine, Aichi, Japan

Article: Ishikawa F, Fukuie T, Matsumoto Y, et al. A two-week, hands-on educational program for primary care pediatricians aimed at equalization of pediatric allergy practice across institutions and regions. JMA J. 2024;7(4):590-9.

Correction 1: Main text, “Educational program” of Materials and Methods section.

Original: In 2019, the contents of AD, asthma, and allergic rhinitis (AR) were added to ensure the management of coexisting allergic diseases, and an additional 36 skill/knowledge objectives and 14 behavioral objectives were set.

Corrected: In 2019, the contents of AD, asthma, and allergic rhinitis (AR) were added to ensure the management of coexisting allergic diseases, and 36 skill/knowledge objectives and 14 behavioral objectives were set.

Correction 2: Main text, “Evaluation of reaction (Kirkpatrick level 1)” of Results section.

Original: Scores for items to be studied in clinical practice, such as patient education programs by physicians (Item No. 4), instructions by nurses (Item No. 5), and experiences of OFC (Item Nos. 6 and 7) were high.

Corrected: 48 participants answered questions Nos. 6a and 7a, and 14 participants answered questions Nos. 6b and 7b. Scores for items to be studied in clinical practice, such as patient education programs by physicians (Item No. 4), instructions by nurses (Item No. 5) were high.

Correction 3: Figure 1.

The correct figure is presented below.

**Figure 1.**
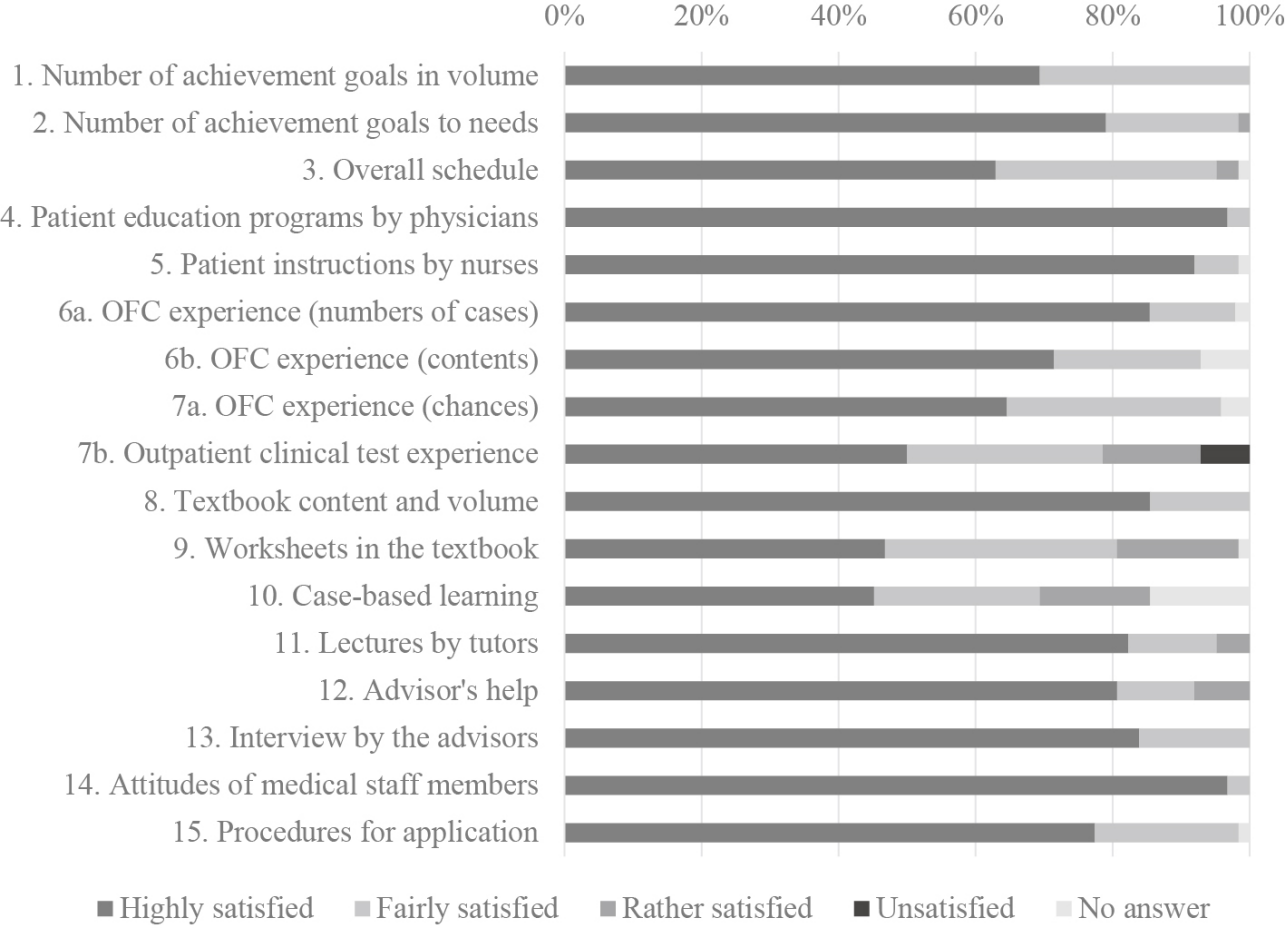


Correction 4: The legends of Figure 1.

Original: Answers for patient education programs by physicians (Item No. 4) and instructions by nurses (Item No. 5) were found to be highly meaningful for the participants.

Corrected: Answers for patient education programs by physicians (Item No. 4) and instructions by nurses (Item No. 5) were found to be highly meaningful for the participants. Questions Nos. 6a and 7a were for the third to seventh term and questions Nos. 6b and 7b were for the eighth to ninth term.

Correction 5: Main text, “Evaluation of knowledge and skills (Kirkpatrick level 2)” of Results section.

Original: Between the eighth and ninth terms, improvements were seen in all items; nevertheless, only two items were statistically significant.

Corrected: Between the eighth and ninth terms, improvements were seen in all items; nevertheless, only four items were statistically significant.

Correction 6: Main text, “Evaluation of behaviors (Kirkpatrick level 3)” of Results section.

Original: Between the third and seventh terms, significant behavioral improvements in six of seven items were shown, except for Item No. 1, which all the respondents achieved already before the course.

Corrected: Between the third and seventh terms, significant behavioral improvements in five of seven items were shown, except for Items Nos. 1 and 2, which many respondents achieved already before the course.

Correction 7: Table 3.

The correct table is presented below. The changes are underlined.

**Table 3.** 

Items related to knowledge and skills (third to seventh terms)		Mean achievement score (SD)		*P* value	Items related to knowledge and skills (eighth to ninth terms)		Mean achievement score (SD)		*P* value
		Pre-training	Post-training				Pre-training	Post-training	
[Diagnosis and evaluation of food allergy]									
1	Diagnosis of immediate allergic response	2.74 (0.64)	3.70 (0.46)	<0.001*	1	Diagnosis of immediate allergic response	2.81 (0.91)	3.44 (0.73)	0.010
2	Explanation of the differences in diagnostic methods for FA	2.36 (0.79)	**3.60 (0.54)**	<0.001*	2	Explanation of the differences in diagnostic methods for FA	2.69 (0.87)	3.56 (0.63)	0.002
3	Interpretation of SPT results	**1.49 (0.80)**	3.38 (0.64)	<0.001*	3	Interpretation of SPT results	2.13 (0.89)	3.44 (0.51)	0.001*
					4	Diagnosis based on allergen components	2.25 (0.86)	3.44 (0.51)	<0.001*
[Oral food challenge test]									
4	Explanation and giving informed consent for OFC	2.39 (1.08)	3.70 (0.51)	<0.001*	5	Explanation and giving informed consent for OFC	2.38 (0.89)	3.13 (0.89)	0.031
5	Prescription and treatment instructions for OFC	2.09 (1.02)	3.60 (0.54)	<0.001*	6	Prescription and treatment instructions for OFC	2.00 (0.97)	2.94 (0.77)	0.003
6	Supervision of preparation by the medical staff for OFC	2.09 (1.04)	3.66 (0.52)	<0.001*	7	Supervision of preparation by the medical staff for OFC	**2.38 (1.15)**	3.38 (0.72)	0.005
7	Preparation of the test food and medical devices for OFC	1.66 (0.81)	3.60 (0.54)	<0.001*	8	Preparation of test food and medical devices for OFC	2.19 (1.05)	3.25 (0.77)	0.002
8	Conducting OFC for a patient	1.66 (0.81)	3.53 (0.55)	<0.001*	9	Conducting OFC for a patient	2.38 (1.09)	3.50 (0.73)	0.002
					10	Conducting double-blind food challenge tests	1.81 (0.91)	2.31 (0.87)	0.015
[Dietary guidance]									
9	Listing notes in reintroducing foods for patients at low risk	2.40 (0.88)	3.49 (0.59)	<0.001*	11	Listing notes in reintroducing foods for patients at low risk	**2.44 (0.89)**	**3.44 (0.63)**	<0.001*
10	Listing notes for sensitized patients	2.15 (0.81)	3.45 (0.54)	<0.001*	12	Listing notes for sensitized patients	2.38 (0.89)	3.31 (0.60)	0.002
11	Guidance on partial introduction or reintroduction	1.87 (0.80)	3.12 (0.65)	<0.001*	13	Guidance on partial introduction or reintroduction	2.31 (0.87)	3.19 (0.91)	0.002
[Emergency treatment]									
12	Immediate allergic response and emergency action	2.77 (0.79)	3.83 (0.38)	<0.001*	14	Immediate allergic response and emergency action	2.94 (0.77)	3.56 (0.63)	0.002
13	Explanation of the efficacy and dosage of AAI	2.68 (0.81)	3.78 (0.42)	<0.001*	15	Explanation of the efficacy and dosage of AAI	3.00 (0.73)	3.69 (0.60)	0.003
14	Rules to the prescription of AAI	2.32 (0.98)	3.54 (0.66)	<0.001*	16	Rules about the prescription of AAI	2.53 (0.99)	3.31 (0.87)	0.004
15	Usage instructions for AAI	2.68 (0.91)	3.81 (0.45)	<0.001*	17	Usage instructions for AAI	3.38 (0.72)	3.69 (0.60)	0.037
[Management of atopic dermatitis]									
16	Description of the loss of skin barrier function, aggravating factors of AD	2.51 (0.80)	3.74 (0.44)	<0.001*	18	Diagnostic criteria of AD	2.56 (0.81)	3.56 (0.51)	0.003
17	Instructing techniques of skincare	2.44 (0.69)	3.79 (0.41)	<0.001*	19	Explanation of the loss of skin barrier function in AD	2.44 (0.81)	3.44 (0.51)	0.002
18	Explanation of the side effects of topical corticosteroids	2.77 (0.60)	3.81 (0.40)	<0.001*	20	Evaluation of the severity of AD	2.25 (0.86)	3.06 (0.57)	0.007
19	Avoiding side effects of topical corticosteroids	2.34 (0.70)	3.72 (0.45)	<0.001*	21	Instructing techniques of skincare	2.63 (0.81)	3.53 (0.64)	0.004
20	Idea of proactive treatment for AD	2.47 (0.95)	3.83 (0.38)	<0.001*	22	Avoiding side effects of topical corticosteroids	2.50 (0.82)	3.53 (0.52)	0.002
					23	Idea of proactive treatment for AD	2.63 (0.96)	3.60 (0.51)	0.005
					24	Exacerbation factors of atopic dermatitis	2.75 (0.68)	3.53 (0.52)	0.004
[Allergy prevention concept]									
21	Knowledge of the theory “dual-allergen exposure hypothesis”	2.30 (0.93)	3.77 (0.43)	<0.001*	25	Knowledge of the theory “dual-allergen exposure hypothesis”	2.00 (0.89)	3.33 (0.49)	<0.001*
[Management of asthma]									
					26	Diagnostic criteria of asthma	2.63 (0.72)	3.40 (0.51)	0.002
					27	Evaluation of the severity and control status of asthma	2.75 (0.68)	3.47 (0.52)	0.002
					28	Exacerbation factors of asthma	2.88 (0.62)	**3.60 (0.51)**	0.002
					29	Evaluation of pulmonary function	2.25 (1.00)	3.33 (0.62)	0.002
					30	Evaluation of fractional exhaled nitric oxide (FeNO)	2.06 (0.93)	2.93 (0.70)	0.002
					31	Conducting airway sensitivity tests	1.19 (0.40)	2.07 (0.70)	0.002
					32	Long-term management of asthma	2.63 (0.72)	3.40 (0.51)	0.006
					33	Guidance on the lifestyle and household environment for preventing asthma exacerbation	2.50 (0.89)	3.40 (0.74)	0.006
					34	Inhalation device	2.56 (1.03)	3.40 (0.63)	0.009
					35	Action plans for acute asthma exacerbation	2.94 (0.85)	3.60 (0.51)	0.005
[Allergic rhinitis]									
					36	Sublingual immunotherapy	2.06 (1.12)	3.20 (0.68)	0.005
[Patient education]									
22	Educational interventions for patients	2.11 (0.68)	3.43 (0.54)	<0.001*

Abbreviations: AAI, adrenaline auto-injector; AD, atopic dermatitis; FA, food allergy; OFC, oral food challenge test; SPT, skin prick test. Note: For the third to seventh terms, *P* < 0.0023 is considered statistically significant and for eighth to ninth terms, and *P* < 0.0014 is considered statistically significant.

Correction 8: Supplementary Table 1.

The correct table is presented below.

**Supplementary Table 1.**