# Supplementary material for: A Two-week, Hands-on Educational Program for Primary Care Pediatricians Aimed at Equalization of Pediatric Allergy Practice across Institutions and Regions
Source: JMA J. 2024 Oct 7;7(4):590–9. doi: 10.31662/jmaj.2024-0127 (PMC11543361; doi:10.31662/jmaj.2024-0127)
Supplement: Supplementary Table 1 [file 2433-3298-7-4-0590-s001.pdf]

Supplemental Table 1. Textual question item of “evaluation of reaction” (third to ninth terms)

1. The number of skill/knowledge goals was appropriate for the course
2. The number of skill/knowledge goals was appropriate for your needs
3. The schedule was neither too tight nor loose
4. Attending patient education programs by physicians was meaningful
5. Attending patient instructions by nurses was meaningful
6. The number of patients undergoing OFC you could oversee was sufficient
7. You could experience the OFC practically (understanding the patient’s medical history, assisting patients, and making decisions on their treatment)
8. The textbook had appropriate content and volume
9. The worksheets in the textbook for taking notes were useful
10. The case-based learning was useful
11. Lectures by tutors helped your learning
12. The advisor helped you participate in the schedule
13. The interview by the advisors during the course was useful
14. The medical staff members were friendly, and it was easy to ask questions
15. The application procedures went smoothly

Abbreviations: OFC, oral food challenge test.
