# Supplementary material for: A Two-week, Hands-on Educational Program for Primary Care Pediatricians Aimed at Equalization of Pediatric Allergy Practice across Institutions and Regions
Source: JMA J. 2024 Oct 7;7(4):590–9. doi: 10.31662/jmaj.2024-0127 (PMC11543361; doi:10.31662/jmaj.2024-0127)
Supplement: Supplementary Table 2 [file 2433-3298-7-4-0590-s002.pdf]

Supplemental Table 2. Textual question item of "evaluation of knowledge and skills" (third to seventh terms)

1. You can diagnose immediate allergic response by confirming patients' medical history
2. You can explain the difference in accuracy between the skin test, specific IgE test (immunoCAP system), and OFC
3. You can reconsider food elimination using the result of the SPT
4. You can explain the indication and procedure of the OFC and obtain the patient's (or their caregiver's) consent
5. You can prepare prescription and treatment instructions for patients undergoing OFC
6. You can supervise medical staff preparation for OFC
7. You can prepare the chart, test food, and medical devices for patients undergoing OFC
8. You can take charge of more than one patient undergoing OFC, observe their symptoms, take records, and treat them
9. You can write notes for patients when reintroducing eliminated foods if they have a low risk for immediate allergic reactions to them
10. You can write notes for patients when continuing or introducing the foods by which they were sensitized
11. You can decide the way of introducing or reintroducing food if the result of the OFC with a small amount of food was negative
12. You can instruct patients and their caregivers on symptoms of immediate allergic response, emergency medications, and the timing they should visit the emergency department
13. You can explain the necessity for AAI, its appropriate dosage (0.15 or 0.3 mg), and when to use it
14. You understand the rules to prescribe AAI, such as the need for obtaining the patient's (or their caregiver's) consent, the physician registration system in Japan, and its costs in the Japanese medical insurance system
15. You can explain how to use AAI using a practice model
16. You can account for the loss of the skin barrier function in patients with AD and its exacerbation factors
17. You can teach skincare techniques to patients with AD and their caregivers
18. You can explain the side effects of topical corticosteroids
19. You can explain how to avoid the side effects of topical corticosteroids
20. You can explain how to maintain the remission state with the idea of proactive treatment for AD
21. You can explain the theory "dual-allergen exposure hypothesis"
22. You can list educational interventions necessary for your patients and their caregivers

Abbreviations: AAI, adrenaline autoinjector; AD, atopic dermatitis; OFC, oral food challenge test; SPT, skin prick test;
