# Supplementary material for: A Two-week, Hands-on Educational Program for Primary Care Pediatricians Aimed at Equalization of Pediatric Allergy Practice across Institutions and Regions
Source: JMA J. 2024 Oct 7;7(4):590–9. doi: 10.31662/jmaj.2024-0127 (PMC11543361; doi:10.31662/jmaj.2024-0127)
Supplement: Supplementary Table 3 [file 2433-3298-7-4-0590-s003.pdf]

Supplemental Table 3. Textual question item of "evaluation of knowledge and skills" (eighth to ninth terms)

1. You can diagnose immediate allergic response by confirming patients' medical history
2. You can explain the difference in accuracy between the skin test, specific IgE test (immunoCAP system), and OFC
3. You can reconsider food elimination using the result of the SPT
4. You can make a diagnosis based on allergen components
5. You can explain the indication and procedure of the OFC and obtain the patient's (or their caregiver's) consent
6. You can prepare prescription and treatment instructions for patients undergoing OFC
7. You can supervise medical staff preparation for OFC
8. You can prepare the chart, test food, and medical devices for patients undergoing OFC
9. You can take charge of more than one patient undergoing OFC, observe their symptoms, take records, and treat them
10. You can assist in conducting double-blind food challenge tests
11. You can write notes for patients when reintroducing eliminated foods if they have a low risk for immediate allergic reactions to them
12. You can write notes for patients when reintroducing eliminated foods that remain at risk for immediate allergic reactions
13. You can propose the method of introducing or reintroducing food if the result of the OFC with a small amount of food was negative
14. You can instruct patients and their caregivers on symptoms of immediate allergic response, emergency medications, and the timing they should visit the emergency department
15. You can explain the necessity for AAI, its appropriate dosage (0.15 or 0.3 mg), and when to use it
16. You understand the rules to prescribe AAI, such as the need for obtaining the patient's (or their caregiver's) consent, the physician registration system in Japan, and its costs in the Japanese medical insurance system
17. You can explain how to use AAI
18. You can explain the diagnostic criteria of AD
19. You can explain the loss of the skin barrier function in patients with AD
20. You can evaluate the severity of AD
21. You can teach skincare techniques of skincare to patients with AD and their caregivers
22. You can explain the drug therapy for AD, possible side effects, and how to avoid side effects
23. You can explain how to maintain the remission state through proactive treatment for AD
24. You can explain the exacerbation factors of AD
25. You can explain the theory of the "dual-allergen exposure hypothesis"
26. You can explain the diagnostic criteria of asthma and its differential diagnosis
27. You can evaluate the severity and control status of asthma
28. You can list exacerbation factors of asthma
29. You can record the flow volume curve correctly and explain the result of the pulmonary function test to your patients (or their caregivers)
30. You can record the fractional exhaled nitric oxide correctly and explain the result to your patients (or their caregivers)
31. You can conduct airway sensitivity tests
32. You can choose an appropriate controller depending on the disease severity
33. You can provide guidance on lifestyle and household environment for preventing asthma exacerbation
34. You can select the inhalation device according to the patient's age and provide guidance on inhalation therapy
35. You can provide guidance on action plans for acute asthma exacerbation to patients and their caregivers
36. You can explain the effects of sublingual immunotherapy, its side effects, and how to take the medicine

Abbreviations: AAI, adrenaline autoinjector; AD, atopic dermatitis; OFC, oral food challenge test.
