# Supplementary material for: A Two-week, Hands-on Educational Program for Primary Care Pediatricians Aimed at Equalization of Pediatric Allergy Practice across Institutions and Regions
Source: JMA J. 2024 Oct 7;7(4):590–9. doi: 10.31662/jmaj.2024-0127 (PMC11543361; doi:10.31662/jmaj.2024-0127)
Supplement: Supplementary Table 4 [file 2433-3298-7-4-0590-s004.pdf]

Supplementary Table 4. Textual question item of “evaluation of clinical practice behaviors” (third to ninth terms)

1. If you see patients under a food-elimination protocol based on the results of positive specific IgE tests, you explain to at least 50% of them and their parents that it is incorrect to make a diagnosis only by specific IgE tests
2. If you see patients under a food-elimination protocol because of uncontrolled AD (even if the reason for consultation was not about allergy), you explain to at least 50% of them and their parents the importance of treating eczema with skincare and prescribed external medicine as soon as possible
3. If you see patients under a food-elimination protocol because of their FA and they have had no severe immediate reactions recently despite positive specific IgE, you perform skin tests or OFC for the reintroduction of foods within 6 months to at least 50% of them
4. If patients with FAs visit your clinic and they have AD, you give practical instructions on skincare and application of external medicine within 3 months for at least 80% of them
5. If you see patients under a strict food-elimination protocol because of previous immediate allergic response or sensitization (even if the reason for consultation was not about allergy) and you think elimination is unnecessary, you provide some interventions within 6 months in at least 50% of them for food reintroduction
6. If patients with FAs visit your clinic and they have difficulty in the reintroduction of foods because of pruritus from AD, you confirm the improvement of their symptoms in 3 months after the intervention in at least 80% of them
7. If patients with FAs visit your clinic and they had a severe immediate allergic response recently, you give information on the action plan including adrenaline autoinjector in at least 80% of them
8. You explain the diagnosis and severity of AD to at least 50% of the patients based on the diagnostic criteria and severity index
9. If you see patients visiting your clinic for AD, you provide plans and guidance on treatment methods based on proactive-remission maintenance therapy
10. In treating AD, you evaluate the exacerbation factors of each patient and provide guidance on lifestyle and household environment to at least 50% of them
11. You apply self-monitoring tools or questionnaires to evaluate the severity and control status of patients with asthma
12. You conduct pulmonary function tests, measurement of exhaled nitric oxide concentration, and airway hypersensitivity test to evaluate the severity and control status of patients with asthma
13. After you introduce inhalation therapy to patients with asthma, you confirm the inhalation procedure
14. You consider indications for sublingual immunotherapy in the treatment of allergic rhinitis and explain its effects, side effects, and how to take the medicine

Question items No.8 to No.14 were for only eighth and ninth terms.

Abbreviations: AD, atopic dermatitis; FA, food allergy; OFC, oral food challenge test.
